# Computational discovery and experimental validation of novel drug targets in immuno-oncology

**DOI:** 10.1186/2051-1426-3-S2-P184

**Published:** 2015-11-04

**Authors:** Ofer Levy, Arthur Machelnkin, Galit Rotman, Amir Toporik, Gady Cojocaro, Liat Dassa, Ilan Vaknin, Spencer Liang, John Hunter, Eyal Neria, Zurit Levin

**Affiliations:** 1 Compugen, Tel Aviv, Israel

## 

The past few years have witnessed a renaissance in the field of immuno-oncology largely due to the clinical success in targeting the immune checkpoints CTLA-4 and PD-1. Towards identification of novel immune checkpoint drug targets we developed a dedicated predictive discovery platform.

The B7/CD28 discovery platform is a predictive model based on genomic and protein features along with expression patterns of known B7/CD28 proteins. The platform has been tested and validated extensively and has demonstrated its validity by identifying non-novel immune checkpoints such as TIGIT and VISTA, which were not used in the design stage.

The B7/CD28 predictive platform was employed to identify several novel immune checkpoint candidates which are currently in different validation stages. In this poster, we will describe our discovery approach as well as our validation path. In addition, we will present experimental data demonstrating the immuno-modulatory function and expression patterns of several of our novel immune checkpoints. These experimental results serve as an additional confirmation to the accuracy of our B7/CD28 predictive discovery platform and shed light on the therapeutic potential of the novel immune checkpoints identified using this unique discovery approach.

